# Asymmetries in Dynamic Valgus Index After Anterior Cruciate Ligament Reconstruction: A Proof-of-Concept Study

**DOI:** 10.3390/ijerph18137047

**Published:** 2021-07-01

**Authors:** Kai-Yu Ho, Andrew Murata

**Affiliations:** Department of Physical Therapy, University of Nevada, Las Vegas, 4505 S. Maryland Parkway, Box 453029, Las Vegas, NV 89154, USA; murata1@unlv.nevada.edu

**Keywords:** anterior cruciate ligament, biomechanics, knee, post-surgical, single-leg hop

## Abstract

Individuals with anterior cruciate ligament reconstruction (ACLR) are at a higher risk for subsequent anterior cruciate ligament (ACL) tears. Risk factors for ACL injuries likely involve a combination of anatomical, biomechanical, and neuromuscular factors. Dynamic knee valgus has been indicated as a possible biomechanical factor for future ACL injuries. Given that knee valgus is often accompanied by contralateral pelvic drop during single-leg activities, a dynamic valgus index (DVI) that quantifies combined kinematics of the knee and hip in the frontal plane has recently been developed. As the premise of asymmetrical DVI between limbs in the ACLR population has not been examined, this cross-sectional study was conducted with the aim to compare DVI between individuals with ACLR and healthy controls. Videos were taken for 12 participants with ACLR and 20 healthy controls when they performed single-leg hopping. One-way ANOVA revealed a higher DVI in the injured limb of the ACLR group when compared to their non-injured limb and to the healthy limb of the control group. As our data showed increased DVI in the injured limb of the ACLR group, the DVI approach accounting for hip and knee kinematics may be used to identify frontal plane movement deficits during single-leg hopping in individuals with ACLR.

## 1. Introduction

Anterior cruciate ligament (ACL) tears are a common orthopedic injury in the general population [1], with increased risk with sports participation and in females [2,3]. Additionally, it has been reported that young athletes who return to sports within 12 months post-ACL reconstruction (ACLR) have a 15 times greater incidence of a second ACL injury when compared to uninjured counterparts [4]. Within 2 years after ACLR, young athletes are still approximately 6 times more likely to sustain a second ACL injury after return to sport [5]. Female athletes’ risk for non-contact ACL injury has been reported to be 3.5 times greater than male athletes [6]. Risk factors for ACL injuries likely involve a combination of anatomical, biomechanical, and neuromuscular factors [7,8]. Anatomic risk factors may include decreased intercondylar femoral notch size, decreased concavity of medial tibial plateau, and increased posterior tibial slope [7,8,9]. Increased knee valgus [8,10,11] and reduced hip and knee flexion [12] during weight-bearing activities are possible biomechanical factors associated with ACL injuries. Neuromuscular timing may also play a role in increased risks for ACL injuries in females as female athletes demonstrate delayed vastus medialis activation during landing, which correlates to increased dynamic knee valgus [13,14].

Excessive dynamic knee valgus is problematic as previous studies have reported it as a possible risk factor for non-contact ACL injury [11,15]. From a tissue mechanics perspective, excessive knee valgus has been found to increase ACL strain [16]. Much of the research on biomechanical risk factors for ACL injuries focuses on the frontal plane knee biomechanics without assessing pelvic motion [15]. However, during single-leg activities, dynamic knee valgus is commonly accompanied by contralateral pelvic drop [17]. Furthermore, hip extensor and hip abductor weakness have been shown to contribute to increased dynamic knee valgus and limited hip flexion during landing [18,19]. Improvements to both femoral and pelvic kinematics in the frontal plane were observed when patients were cued to decrease their knee valgus during a single-leg squat [17]. These findings highlight the values of assessing frontal plane kinematics of both the knee and hip when evaluating individuals at risk for ACL injuries.

The dynamic valgus index (DVI) is a recently developed method that is used to quantify the combined two-dimensional (2D) kinematics of the knee and hip in the frontal plane [20]. DVI may be a better indicator of the kinematical faults in the lower extremities for those at risk of ACL injuries because it provides a more extensive analysis of the hip and knee joint motions contributing to dynamic knee valgus [20]. However, while DVI has been shown to be a valid and reliable measure from Scholtes and Salsich’s work, it was originally assessed on a single-leg squat test in individuals with patellofemoral pain [20]. Scholtes and Salsich [20] stressed the need for further research to examine DVI with other tasks and populations. As individuals with ACLR can exhibit asymmetrical dynamic knee valgus even at 2 years post-surgery [21], it is important to assess their frontal plane lower-extremity kinematics during single-leg activities as well. To date, even though individuals with ACLR are thought to have an increased risk of another ACL injury [5] from asymmetries between limbs [21,22], DVI has not been assessed in persons with ACLR. The purpose of this study was to compare the DVI during landing in a single-leg hop test between the injured and non-injured limbs of individuals with ACLR and the healthy limb of healthy controls. We hypothesized that there would be a higher DVI during a single-leg hop test in the injured limb of individuals with ACLR when compared to the non-injured limb of individuals with ACLR and the healthy limb of the control group.

## 2. Materials and Methods

### 2.1. Participants

A cross-sectional study design was used for this work. The data from an existing study were used to estimate the sample size for detecting differences in frontal plane lower-extremity kinematics between groups [23]. With 95% power, an α level of 0.05, and a calculated effect size of 1.78, we estimated that 8 individuals per group would be needed. However, due to the exploratory nature of this study, efforts were made to recruit more participants in both ACLR and control groups in the Las Vegas area during the study period (between 2017 and 2018).

The participants in the ACLR group were included if they (1) were 18–45 years, (2) had a non-contact, unilateral ACL injury with a surgical repair within the past 6 months to 5 years, (3) had approval to return to sports by their surgeon/physician, and (4) scored a minimum of 60% on the International Knee Documentation Committee (IKDC) Subjective Knee Evaluation form. Individuals with ACLR were excluded if they reported additional ligamentous injury or were pregnant. As ACL injuries are often accompanied by meniscal damage, concomitant meniscal injuries requiring surgery were allowed in our study [24,25,26]. Control participants were included if they (1) were 18–45 years and (2) reported a minimum of a 60% score on the IKDC Subjective Knee Evaluation form. Participants in the control group were disqualified if they were pregnant or reported any history of lower-extremity surgery or major musculoskeletal injury. The IKDC Subjective Knee Evaluation form is a reliable measure that was used to assess current function and symptoms of the knee [27]. The cut-off score of 60% was based on normative data [28] to confirm that the participant’s knee function was good enough to perform the required task.

Prior to participation, participants provided informed consent approved by the institutional review board of University of Nevada, Las Vegas (IRB# 913605). No changes were made to the approved methodology (including participant eligibility) after the study commenced.

### 2.2. Procedures

Each participant attended one session of data collection. Data collection took place at the Biomechanics Core Laboratory at University of Nevada, Las Vegas. The videos for the 2D analysis were recorded at 30 frames per second on an iPad Air 2 tablet (Apple Inc., Cupertino, CA, USA). A tripod fixed at 359 cm from the landing area and 35 cm above the ground was used to mount the iPad to capture frontal plane kinematics.

Participants received verbal instructions and a demonstration before they were allowed to perform their practice repetitions. Specifically, participants were directed to hop as far as they were able to. Participants were allowed to perform a maximum of two practice repetitions to become familiar with the task, which is consistent with the procedure reported in the literature [29]. Single-leg hop testing was chosen because it is a recommended test for determining post-operational function in individuals with ACLR [30].

After completion of the practice repetitions, participants were asked to successfully perform the task 3 times, with a maximum of 10 attempts allowed to avoid fatigue. A successful attempt required maintaining balance upon landing for 3 s without shifting the landing foot. For the participants with ACLR, the non-injured leg was tested first. For the participants in the control group, the dominant leg (determined by the leg used for landing from jumping) was tested.

### 2.3. Data Processing

Two-dimensional lower-extremity kinematics were measured in the frontal plane using Kinovea software, which has been used to assess dynamic knee valgus previously [13,31]. Hip frontal plane projection angle (FPPA), knee FPPA, and DVI were determined for each trial at maximum knee flexion during landing. The average values of the 3 trials for hip FPPA, knee FPPA, and DVI were calculated for statistical analyses. All the measurements were performed by the same investigator, who was blinded to information about the injured/non-injured side of the participants in the ACLR group.

**Figure 1:**
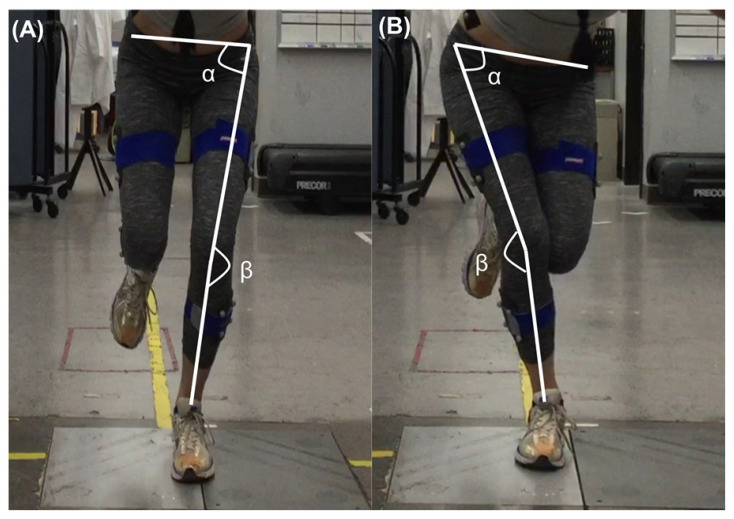
Measurements of hip FPPA, knee FPPA, and DVI for (**A**) non-injured limb and (**B**) injured limb in a participant with ACLR. Hip FPPA is defined as 90° minus the angle between the pelvis segment and the thigh segment (i.e., 90° − α). Knee FPPA is defined as 180° minus the angle between the thigh segment and the shank segment (i.e., 180° − β). DVI is defined as the sum of knee FPPA and hip FPPA. Abbreviations: FPPA, frontal plane projection angle; DVI, dynamic valgus index.

Hip and knee FPPAs were obtained with the following definitions of the pelvis, thigh, and shank segments [20]. The first line was placed between the anterior superior iliac spines to represent the pelvis. Another line was drawn from the center of the knee joint through the middle of the thigh to represent the thigh. The third line was drawn from the center of the knee joint to the center of the ankle joint to represent the shank. Hip FPPA was calculated by subtracting the angle between the pelvis and the thigh from 90° (Figure 1). Higher values for the hip FPPAs represented greater hip adduction. Knee FPPA was calculated by subtracting the angle between the thigh and the shank from 180° (Figure 1). Higher values for the knee FPPAs demonstrated greater knee valgus. A resultant DVI was established by calculating the sum of the hip and knee FPPAs (Figure 1).

### 2.4. Statistical Analysis

All statistical analyses were performed with the use of SPSS 24.0 statistical software (International Business Machines Corp, Armonk, New York, NY, USA). The intraclass correlation coefficient (ICC) was used to determine test–retest reliability for the measurements of hip FPPA, knee FPPA, and DVI. ICC values were interpreted according to the following criteria: poor < 0.04, fair 0.4 to 0.7, good 0.7 to 0.9, and excellent > 0.9 [32]. The participants’ characteristics (age, weight, and height) and the movement measures (knee FPPA, hip FPPA, and DVI) were assessed for normality using Shapiro–Wilk tests and found to be normally distributed. Independent t tests were used to assess the differences in age, weight, and height between individuals with ACLR and healthy controls. One-way ANOVA with post-hoc analyses were performed to compare knee FPPA, hip FPPA, and DVI between the injured and non-injured limbs of the ACLR group and the healthy limb of the control group. A significant difference was defined as a *p*-value less than 0.05.

## 3. Results

### 3.1. Participant Characteristics

Twelve participants (10 females and 2 males; age: 24.5 ± 7.2 years; height: 164.2 ± 11.2 cm; weight: 67.9 ± 8.2 kg) with history of ACLR (time since surgery: 2.4 ± 1.4 years) and twenty healthy controls (7 females and 13 males; age: 25.2 ± 2.8 years; height: 175.1 ± 7.5 cm; weight: 72.9 ± 10.4 kg) participated in this study. Both groups showed similar age (*p* = 0.752) and weight (*p* = 0.164), while the height of the control group was significantly greater than that of the group with ACLR (*p* = 0.008).

### 3.2. Measurement Reliability

To obtain the reliability for knee FPPA, hip FPPA, and DVI, the investigator performed repeated measurements on 5 participants’ videos on 2 separate occasions (at least one week apart). The intra-rater reliability was found to be excellent, with an ICC score of 0.92, 0.92, and 0.93 for knee FPPA, hip FPPA, and DVI, respectively.

### 3.3. DVI, Knee FPPA, and Hip FPPA

**Figure 2:**
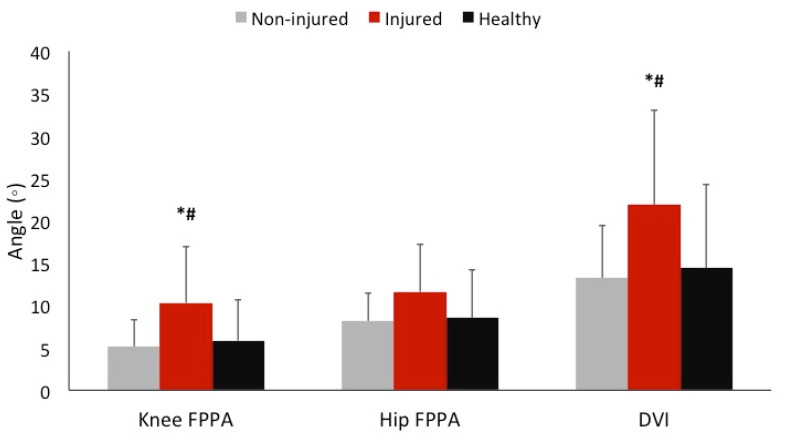
Comparisons of knee FPPA, hip FPPA, and DVI between the non-injured and injured limbs of participants with ACLR and the healthy limb of healthy participants. * Indicates a statistically significant difference from the non-injured limb, # indicates a statistically significant difference from the healthy limb. Abbreviations: FPPA, frontal plane projection angle; DVI, dynamic valgus index.

The ANOVA revealed a statistically significant difference in DVI during single-leg hopping (*p* = 0.049). The post-hoc analyses showed that DVI was significantly higher in the injured limb of the ACLR group when compared to the non-injured limb of the ACLR group (*p* = 0.035) and to the healthy limb of the control group (*p* = 0.031). No difference was found in DVI between the non-injured limb of the ACLR group and the healthy limb of the control group (*p* = 0.744) (Table 1; Figure 2).

The ANOVA revealed a statistically significant difference in knee FPPA during single-leg hopping (*p* = 0.041). The post-hoc analyses showed that knee FPPA was significantly greater in the injured limb of the ACLR group when compared to the non-injured limb of the ACLR group (*p* = 0.029) and to the healthy limb of the control group (*p* = 0.027). There was not a difference in knee FPPA between the non-injured limb of the ACLR group and the healthy limb of the control group (*p* = 0.720) (Table 1; Figure 2).

Lastly, the ANOVA showed that there was not a statistically significant difference in hip FPPA during single-leg hopping between the injured limb and non-injured limb of the ACLR group, and the healthy limb of the control group (*p* = 0.127) (Table 1; Figure 2).

## 4. Discussion

The purpose of this study was to compare DVI during landing in a single-leg hop test between the injured and non-injured limbs of individuals with ACLR and the healthy limb of healthy controls. In support of our hypotheses, our results showed that DVI was significantly higher during a single-leg hop test in the injured limb of individuals with ACLR when compared to the non-injured limb of the same individuals and to the healthy limb of control participants. We also observed a higher knee FPPA during a single-leg hop test in the injured limb of individuals with ACLR. Additionally, the between side difference in the DVI composite measure was higher than the knee FPPA alone, suggesting that DVI may provide a more in-depth metric than knee valgus angles alone. While DVI is a newly developed method that has been shown to be valid and reliable in individuals with patellofemoral pain [20], to our knowledge, this is the first study to examine the DVI during single-leg hopping in individuals with ACLR.

Our results parallel those of the prior DVI study by Scholtes and Salsich [20] in that we found significantly greater knee FPPA and DVI when comparing the injured limb to the non-injured limb or the healthy limb of controls during functional activities. However, while there is a trend towards a larger hip FPPA in the injured limb of our cohort with ACLR as compared to the non-injured limb of the participants with ACLR and the healthy limb of the control participants (*p* = 0.127), the difference between limbs did not reach a statistically significant difference. This may be attributed to the relatively smaller sample size employed in our research and the large standard deviation observed in this variable. The greater variability in hip FPPA observed in our work may be attributed to the fact that both female and male participants were recruited in our work, while only female participants were studied in the work of Scholtes and Salsich [20].

Recent evidence suggests that knee valgus alone may not be predictive of ACL injury or re-injury [33]. As evidence has shown that deficits in frontal plane hip mechanics and postural stability may contribute to second ACL injury risk [34], the inclusion of hip joint kinematics may be a better predictor of ACL rupture risk than analysis of knee joint kinematics alone. A recent study by Peebles et al. [35] found reduced symmetry in individuals with ACLR compared to controls, but found no between-group difference in knee FPPA in a bilateral landing. However, their work revealed a significant difference in knee kinematics during unilateral landing [35]. Based on our data and current literature, it is suggested that unilateral landing tasks may be more proficient at detecting asymmetries and that there may be a need to assess both the hip and the knee for asymmetries between limbs of individuals with ACLR.

Furthermore, recent systematic reviews and clinical practice guidelines suggest that the increased risk of future ACL injury following an ACLR may be due to altered neuromuscular function and biomechanics, such as greater hip internal rotation and dynamic knee valgus [36,37,38]. Despite the potential for altered biomechanics in contributing to increased risk of ACL injury, current clinical practice guidelines indicate that current studies are lacking in both objective physiological criteria for return to play post-ACLR and a test battery that can accurately predict risk of re-injury in athletes [37]. A recent study also highlights the need for obtaining the pre-operative limb asymmetry index, which has been found to be more predictive of a second ACL injury than the post-operative limb asymmetry index [22]. Thus, the clinical implication of DVI in rehabilitation/sports medicine settings is to potentially utilize the DVI approach as an assessment tool, in conjunction with hip and knee strength testing and hop testing, to identify asymmetries of both the hip and knee during a functional task. Further longitudinal studies are needed to measure the DVI pre- and post-ACLR to better understand the predictive validity of DVI measurements for return to play and ACL injury recurrence. In addition, DVI assessment could potentially contribute to ACL injury prevention programs as neuromuscular and proprioception programs have been shown to reduce ACL injury risk by 50.7%, particularly in female athletes [39]. Neuromuscular warm-up exercise has been shown to immediately increase pre-activation of knee stabilizer muscles, which could potentially decrease ACL load and dynamic valgus [40]. Since late neuromuscular timing, specifically of the vastus medialis, has been correlated to increased dynamic knee valgus [13,14], incorporation of the DVI assessment with neuromuscular training could potentially provide a more in-depth measure of the effects of this training.

The results presented above should be viewed in light of several limitations. First, given that a cross-sectional design was used in the study, it remains unclear if the movement deficits observed in the injured limb occurred before or after ACLR. As such, it cannot be confirmed that asymmetries in DVI and knee FPPA during single-leg hopping were the result of ACLR in this cohort. In addition, 2D motion data collection may be less accurate than 3D measurements, leading to increased errors in the measurements of lower-extremity kinematics during dynamic activities. However, due to consistent camera positioning and data collection, that error may be mitigated as it can be considered similar across all participants. Additionally, while the information about the injured/non-injured side of the participants in the ACLR group was blinded to the evaluator responsible for outcome measures, the participants’ group allocation information was not blinded. Another limitation of the study is that the results may not be generalizable to other tasks or populations as this study included individuals with ACLR and examined single-leg hop testing only. Furthermore, sex distribution was different between the control group (13 males: 7 females) and the ACLR group (2 males: 10 females), which resulted in the body height difference between groups and could potentially contribute to the different frontal plane kinematics of the hip and knee seen between groups. ACL injuries have been found to be more prevalent in females [2], and it has been suggested that females exhibit higher hip adduction and knee abduction during single-leg squatting [41]. In our work, while the movement measures between sexes were not different in any of the 3 limbs from t tests, such findings may be attributed to the small sample size employed. Taken together, although our study contained the necessary participant numbers to meet the pre-calculated sample size, a future larger-scale study that incorporates similar sex distribution in individuals with and without ACLR is critical for investigating the potential sex differences in DVI in individuals with ACLR and healthy controls. Future research should also explore the DVI in other orthopedic conditions and functional tasks, with additional efforts to optimize the study designs (e.g., blind procedures).

## 5. Conclusions

The DVI approach that takes into consideration both hip and knee kinematics in the frontal plane may be useful in identifying frontal-plane movement deficits during single-leg hopping in individuals with ACLR.

## Figures and Tables

**Table 1:** Comparisons of knee FPPA, hip FPPA, and DVI between the non-injured and injured limbs of participants with ACLR and the healthy limb of healthy participants.

		Injured	Non-Injured	Healthy
Knee FPPA (°)	Female	11.0 ± 6.9	5.5 ± 3.3	7.6 ± 8.3
	Male	6.0 ± 1.4	3.3 ± 2.8	4.4 ± 5.5
	All	10.3 ± 6.6 *#	5.1 ± 3.2	5.8 ± 4.8
Hip FPPA (°)	Female	12.0 ± 5.9	8.3 ± 3.7	9.4 ± 5.8
	Male	9.0 ± 0.9	7.5 ± 2.6	8.0 ± 5.4
	All	11.6 ± 5.6	8.1 ± 3.4	8.6 ± 5.7
DVI (°)	Female	23.0 ± 11.6	13.8 ± 6.6	17.0 ± 13.7
	Male	15.0 ± 2.4	10.8 ± 5.4	12.4 ± 10.2
	All	21.9 ± 11.1 *#	13.2 ± 6.2	13.8 ± 11.2

* Indicates a statistically significant difference from the non-injured limb, # indicates a statistically significant difference from the healthy limb. Abbreviations: FPPA, frontal plane projection angle; DVI, dynamic valgus index.

## Data Availability

The data presented in this study are available upon request from the corresponding author.
